# Intervene to Improve: Awareness of Testicular Self-Examination and Testicular Cancer Among Male Patients at a Tertiary Care Hospital in Lahore, Pakistan

**DOI:** 10.7759/cureus.33838

**Published:** 2023-01-16

**Authors:** Muazzam Waheed, Muhammad Shaheer Luqman, Ubaid Ullah Bhatti, Haseeb Mehmood Qadri, Hasan Saeed, Muhammad Saad Babar, Muhammad Sheraz, Saad Abdullah, Muhammad Awais Ahmad, Ali Munawar

**Affiliations:** 1 General Surgery, Lahore General Hospital, Lahore, PAK; 2 General Surgery, Jinnah Hospital, Lahore, PAK; 3 General Surgery, Allama Iqbal Medical College, Lahore, PAK; 4 General Surgery, Shaikh Zayed Hospital, Lahore, PAK

**Keywords:** cryptorchidism, male sexual health, prevention, onco-urology, awareness of general population, self-testicular examination, testis cancer

## Abstract

Background and objective

Testicular cancer is the commonest of all types of cancer males are affected with. Testicular cancer, when diagnosed early, has one of the best prognoses. However, in Pakistan, early detection is hindered by religious and sociocultural norms, lack of education, and awareness deficit. Testicular self-examination (TSE) can significantly facilitate early detection of the condition and decrease associated mortality rate. This study aimed to acquire the frame of mind regarding testicular cancer (TC) and testicular self-examination (TSE) among the male outdoor patients of Lahore General Hospital, Lahore.

Materials and methods

After ethical considerations, elaborated literature review and consequent pilot study were done to develop a bilingual questionnaire. Following patient consent, answers to a set of questions were noted down by the authors. A 90-second bilingual, motivational video was displayed and an educational pamphlet on the same topic was also handed over. Afterward, another survey was conducted to grasp the comprehension, satisfaction, and willingness to spread the message.

Results

About 92% of the subjects had not heard of or performed TSE and 58.3% mentioned lack of education as the reason for not knowing the method. Eighty-two percent patients had never heard of TC. Post-education, 100% patients claimed that their knowledge of the subject improved and 97% were ready to teach other male relatives.

Conclusion

The results indicate that the population’s lack of awareness regarding testicular self-examination and testicular cancer is alarming. Most subjects did not know the age group, risk factors, presentation, and early prevention of testicular cancer.

## Introduction

Testicular cancer (TC) makes up 1% of all types of cancer. It, however, is the most common type of cancer among young males, i.e., aged 15-35 years, with a 14% mortality rate of all types of cancer in this age group [1]. As testicular cancer occurs in early life and due to its ability to be treated successfully, this category of cancer can have a crucial impact on the physical and psychological aspects of life of a male being. They also have the greatest impact on the loss of years of life for this population [1].

Within our community, significant attention is given to female health issues, and male health issues are often overlooked which also require significant attention. Internationally, it is recognized that males are disinclined to pursue medical help, procrastinating till symptoms become incapacitating. Such attitude may be because of traditional male gender socialization and social values that put male health at risk, the stigma of fragility associated with seeking help, or lack of awareness regarding the risk factors and warning signs of diseases such as testicular cancer [2].

Various risk factors have been implicated in the pathogenesis of TC, the most common being cryptorchidism, i.e., undescended testes, having 1% incidence [3]. Other risk factors include family history, trauma, genitourinary system abnormalities, and endocrine dysfunctions [4]. The risk of developing neoplasm in the second testicle, after diagnosis of cancer in the first testicle, increases 700 times as compared to the general population [3].

As with other cases of cancers, early detection of TC strongly affects prognosis. The knowledge about earliest signs and timely detection can determine better prognosis and impact a number of lives. The earliest detectable clinical feature is hard, small painless lump in the testicle. Pain is a late feature [5]. Testicular cancer has a cure rate of 96% when diagnosed in the early stages and only 20-50% when diagnosed in the late stages [4]. Pakistan is a developing country where one-third of the population is living in poverty and hence cannot afford costly nationwide diagnostic procedures [6]. The American Cancer Society (ACS) recommends testicular self-examination (TSE) as a cost-free and effective method for TC diagnosis. Unfortunately, TSE is hardly taught and minimally practiced in medicine as confirmed by a recent study [7]. Another study made a case for carrying out TSE using a cost-benefit analysis, concluding that a 2.4:1 cost-to-benefit ratio exists for a case of testicular cancer caught in early stages versus a case diagnosed in advanced stages. Hence, TSE is highly indicated to pick TC at its initial stages leading to greater chances of survival and decreased disease burden worldwide [4].

This study aimed to obtain baseline levels of knowledge, awareness, and attitudes regarding testicular cancer (TC) and testicular self-examination (TSE) among male patients of outpatient department (OPD) of Lahore General Hospital, Lahore, Pakistan. We try to educate our patients and assess their post-intervention knowledge and attitude toward this sensitive subject. To our best knowledge and detailed literature search using PubMed, Scopus, and Google Scholar, this is the second study on this topic from our country assessing the perception of common men.

## Materials and methods

After approval from the Ethical Review Board (ERB) of Lahore General Hospital, Lahore, Pakistan, with reference #38/22, this descriptive cross-sectional study was conducted from April 01, 2022, to June 30, 2022, on the out-patient clinic days of Department of General Surgery, Unit-II, Lahore General Hospital, Lahore, Pakistan, in which the visiting patients were randomly selected as per their consent.

Pre-intervention assessment

After detailed literature search and a pilot study on 10 patients, we developed a bilingual questionnaire via Google Docs, both in English and Urdu languages for assessing the myths, thoughts, and awareness of patients regarding testicular self-examination and testicular cancer. The pro forma included questions under five headings, i.e., awareness of testicular self-examination (TSE), awareness of testicular cancer (TC), thoughts and myths about TC, quality of knowledge and satisfaction, and intention of testicular self-examination. Informed consent was the vital prerequisite of each session lasting 10 minutes roughly per participant.

The intervention

The authors developed an awareness-based pamphlet on TC and TSE (Appendix 1) [8]. We used an educational video on “how to examine your testicles?” that was developed by the Hirslanden Private Hospital Group available on YouTube (Appendix 2) [9]. We had acquired copyright permission from the mentioned authority to develop the dubbed version of their content in the Urdu language as it was readily available in the English language. After securing due responses of each patient, this awareness-based pamphlet was handed over to each patient followed by the display of aforementioned 88-second video clip in the native language.

Post-intervention feedback

We surveyed each patient post-intervention to enquire about their understanding, satisfaction with the quality of survey, and willingness to teach others about the subject matter. After data collection on Microsoft Excel, a statistician compiled the results.

## Results

**Figure 1:**
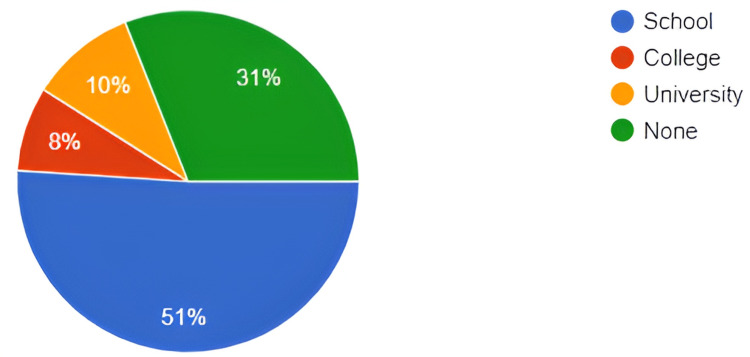
Levels of education of studied sample.

The population taken in this study consisted of 124 males only, as this is a gender-specific health issue. The population individuals are divided on the basis of their respective levels of education and it is statistically explained in the pie diagram (Figure 1).

About 92% of the subjects had not heard of or performed TSE and 58.3% mentioned lack of education as the reason for not knowing the method, while 74% were agreeing simultaneously that TSE can be vital to detect testicular diseases at an early stage (Table 1).

**Table 1:** Awareness of testicular self-examination (TSE).

S. no.	Question	Options	Percentage
1.	Have you heard of testicular self-examination?	Yes	8%
		No	92%
2.	Have you requested to get information about testicular self-examination?	Yes	3%
		No	97%
3.	Have you performed testicular self-examination?	Yes	8%
		No	92%
4.	If yes, what is the frequency of testicular self-examination?	A few times last year	45.5%
		A few times in the last six months	45.4%
		Once a month	9.1%
5.	If no, what is the most likely reason for not doing testicular self-examination?	Not knowing the examination	58.3%
		Not caring	30.1%
		Fear of the worst result	3%
		Think examination is a sin and feel guilty	8.6%
6.	Do you think testicular self-examination is vital to detect testicular diseases at an early stage?	Yes	74%
		No	26%
7.	Testicular self-examination should be done in the shower or shortly after the shower.	Yes	32%
		No	3%
		Don’t know	65%
8.	Testicular self-examination should be done regularly every month.	Yes	24%
		No	7%
		Don’t know	69%

Eighty-two percent participants had never heard of TC and 93% were ignorant of the fact that it is most commonly seen in males aged 15-35 years. Results from this section of the survey revealed an extreme lack of awareness about TC (Table 2).

**Table 2:** Awareness of testicular cancer (TC).

S. no.	Questions	Options	Percentage
1.	Have you heard of testicular cancer?	Yes	18%
		No	82%
2.	Have you ever requested to get information regarding testicular cancer?	Yes	4%
		No	96%
3.	Testicular Cancer is most commonly seen in 15-35 aged male groups.	True	4%
		False	3%
		Don’t know	93%
4.	The most important risk group for testicular cancer is ones with undescended testes.	True	7%
		False	1%
		Don’t know	92%
5.	Chance of recovery increases by 80-90% with early diagnosis.	True	42%
		False	8%
		Don’t know	56%
6.	The earliest diagnostic method for testicular cancer is self-examination.	True	31%
		False	4%
		Don’t know	65%
7.	Testicular cancer can be prevented as a palpable lump, swollen testes, or heaviness in the testes.	True	27%
		False	1%
		Don’t know	72%

Among 124 patients, 100% claimed that their knowledge of the subject improved post-intervention and 99% of them were satisfied with the quality of the survey. When asked about how soon they will conduct testicular self-examination, 76% of patients agreed to do it as soon as possible, and 20% within this month, while 4% patients denied doing self-examination. About 97% of individuals agreed to share the knowledge with their male members of family and friends.

## Discussion

The World Health Organization (WHO) has recommended education to promote early diagnosis and prevention of early signs of cancer by self-organ examination. It is suggested that testicular self-examination (TSE) is an essential intervention in the early diagnosis of testicular cancer (TC) [6]. This study was done to assess the knowledge of general outdoor patients regarding TSE and TC. In our awareness-based campaign, 92% of the subjects in this study had no knowledge regarding testicular self-examination and the correct way of performing it in accordance with the recommended guidelines. This corresponds with international research findings shown by the studies of Irani, Turkish, Nigerian, and American doctors that 90%, 91%, 90.66%, and 79% of total male participants tend to lack knowledge about TSE, respectively [1,7,10,11]. However, another Pakistani study documents that 96% participants had no knowledge about TSE [6].

In our research, only 8% of men knew how to perform TSE. This percentage is relatively high worldwide. A multi-ethnic study by Khadra et al. demonstrates that 49% of participants knew the method of TSE and had performed it in the past year [12]. Similarly, a Polish study reveals that >50% medical school students never performed TSE [13].

Fifty-eight percent of the subjects from our study stated the main reason for the lack of performing TSE is lack of education. This result is comparable with the findings of another study showing 71% of non-performers of TSE had actually no knowledge of its method and significance. Still another Bahraini study highlights lack of education as the most common reason for inability to perform TSE among 73% individuals [10,14]. At the same time, 8.6% participants were hesitant due to religious norms, in contrast to a previous study that suggests sociocultural and religious norms and fear of detecting cancerous lumps could affect most subjects [7]. Despite a lack of knowledge, 84% believed that TSE is a vital tool to detect TC at an early stage, as shown in previous studies [14].

The American Cancer Society has recommended performing TSE monthly [1]. Approximately, 65% of males interviewed did not know that TSE should be done in the shower or shortly after. Eighty-two percent of subjects in our study said that they had not heard about TC, likewise, other studies conducted in other regions showed similar low percentages [2,7,10,14,15]. There was no definitive knowledge about the prevalence of testicular cancer among various age groups in majority of the subjects under consideration, risk factors, early presentation, and tools for early prevention. All of these findings are consistent with previous literature on awareness, knowledge, and attitudes toward TC [2,13-15].

After educating the importance of TSE, 96% were optimistic about doing it themselves either this month or in a timeframe similar to other studies and 97% agreed to share the knowledge further as instructed [7,14]. This aligns with an international survey that identifies men who had been taught how to perform TSE by a health professional were more comfortable discussing any abnormality [2].

The authors acknowledge the limitations of this study. The fact that it is a single-center audit with a small sample size is a limiting factor. We cannot generalize our results for the inhabitants of any specific area. We will consider enhancement in the sample size in future to further elaborate on the importance of this topic. Particular populations can also be segregated for the same topic like medical professionals, teenagers, and adults, in future.

## Conclusions

Worldwide studies have revealed that knowledge of TC, along with the practice of TSE among the general population, is not satisfying. These inadequate results could be attributed to the lack of education, religious and social norms, or fear of detecting a lump. While testicular carcinoma remains the most common type of tumor in young adults, the general public is unaware of its causes, risk factors, and self-examination techniques to a large extent, as signified by our research.

There is a need for the government and health institutes to undergo such steps that educate the males regarding these diseases to minimize the risk factors and embedding of testicular self-evaluation techniques for early diagnosis. Therefore, healthcare providers should raise awareness of TC and change the population's attitude regarding all misconceptions, i.e., signs and symptoms, as well as the importance of performing testicular self-examination, which plays a critical factor in early diagnosis. Government should make adequate health policies regarding the importance of TC and performing TSE regularly with immediate effects addressing the utilization of modern technology and including awareness in the primary education curriculum. Awareness ads via television screens and social media platforms are an established mode of communicating the message. Discussions can be conducted at local religious centers, with the assistance of leaders of various creeds and faiths. Separate male sexual health clinics are the dire need of the hour, where males can mention their concerns and discuss their problems openly. These steps may decrease the incidence of testicular carcinomas resulting in a better quality of life and lower distress in health institutes.

## Figures and Tables

**Figure 2:**
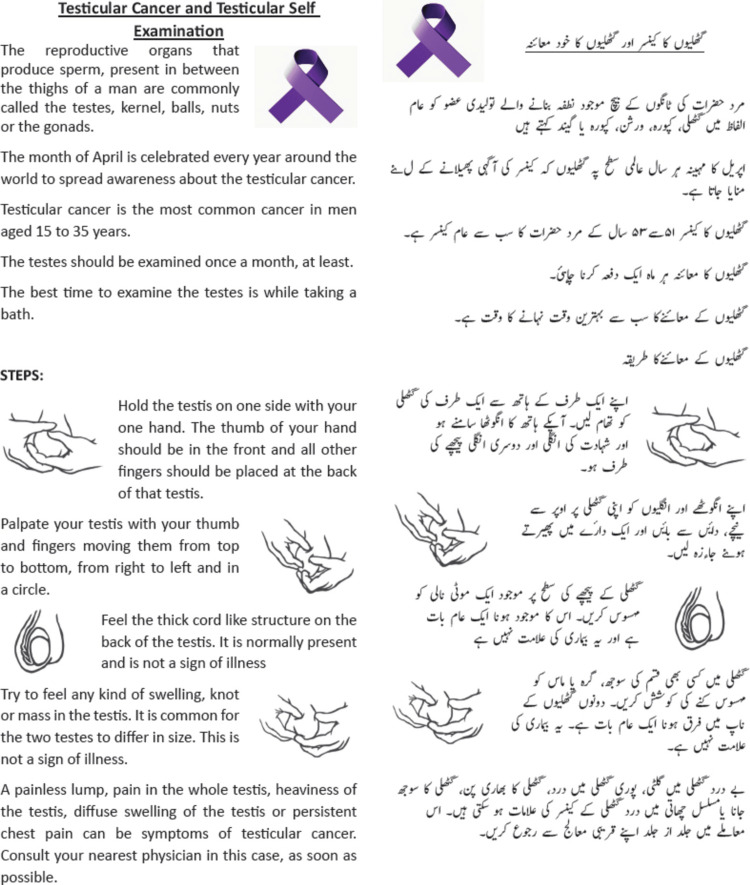
Will men tie their violet ribbons? Testicular cancer and testicular self-examination. This is author's original work published in another journal [8].

**Video 1:** How to correctly examine your testicles - Hirslanden Private Hospital Group. Special permission has been taken from Dr. Med. Claude Kaufmann, Head Media Relations and Corporate Newsroom, Hirslanden Private Hospital Group.

## Appendices

Appendix 1

Appendix 2
